# Transferable Hirshfeld atom model for rapid evaluation of aspherical atomic form factors

**DOI:** 10.1107/S2052252524001507

**Published:** 2024-03-01

**Authors:** Michał Chodkiewicz, Leonid Patrikeev, Sylwia Pawlędzio, Krzysztof Woźniak

**Affiliations:** a Biological and Chemical Research Centre, Department of Chemistry, University of Warsaw, Żwirki i Wigury 101, Warszawa 02-089, Poland; b Neutron Scattering Division, Oak Ridge National Laboratory, Oak Ridge, TN 37831, USA; Formby, Liverpool, United Kingdom

**Keywords:** transferable Hirshfeld atom model, quantum crystallography, aspherical atom refinement, TAAM, HAR

## Abstract

Form factors based on aspherical models of atomic electron density have brought great improvement in the accuracies of hydrogen atom parameters derived from X-ray crystal structure refinement. Today, two main groups of such models are available, the banks of transferable atomic densities parametrized using the Hansen–Coppens multipole model which allows for rapid evaluation of atomic form factors and Hirshfeld atom refinement (HAR)-related methods which are usually more accurate but also slower. In this work, a model that combines the ideas utilized in the two approaches is tested. It uses atomic electron densities based on Hirshfeld partitions of electron densities, which are precalculated and stored in a databank. This model was also applied during the refinement of the structures of five small molecules. A comparison of the resulting hydrogen atom parameters with those derived from neutron diffraction data indicates that they are more accurate than those obtained with the Hansen–Coppens based databank, and only slightly less accurate than those obtained with a version of HAR that neglects the crystal environment. The advantage of using HAR becomes more noticeable when the effects of the environment are included. To speed up calculations, atomic densities were represented by multipole expansion with spherical harmonics up to *l* = 7, which used numerical radial functions (a different approach to that applied in the Hansen–Coppens model). Calculations of atomic form factors for the small protein crambin (at 0.73 Å resolution) took only 68 s using 12 CPU cores.

## Abstract 2

A databank of atomic densities calculated using Hirshfeld partition has been developed which allows for refinement with similar accuracy to Hirshfeld atom refinement without the need for time-consuming wavefunction calculations.

## 1. Introduction

Most crystallographic refinements against X-ray data rely on spherical atomic electron density models, *i.e.* the independent atom model (IAM) which leads to poor determination of structural parameters for hydrogen atoms. The accuracy of their determination greatly improves when aspherical atomic densities are employed. The availability of methods involving aspherical models of atomic electron densities has greatly increased with the development of quantum crystallography in recent years. The methods applicable for accurate X-ray refinement of structural parameters for hydrogen atoms can be roughly divided into two groups: methods using transferable atomic densities parameterized with the Hansen–Coppens multipole model (HCMM; Hansen & Coppens, 1978 ▸) and methods related to Hirshfeld atom refinement (HAR; Jayatilaka & Dittrich, 2008 ▸; Capelli *et al.*, 2014 ▸).

Although the HCMM allows for experimental determination of electron densities, it is not well suited for the refinement of hydrogen atom structural parameters owing to their correlation with charge density parameters. This problem can be circumvented by fixing charge density parameters and refining only structural parameters. It was observed that HCMM parameters for atoms in a similar chemical environments were also similar and can be transferred between chemically similar atoms. It led to the development of ELMAM, a databank of transferable atomic densities (Pichon-Pesme *et al.*, 1995 ▸). Subsequently, a number of such databanks have been developed: ELMAM2 (Zarychta *et al.*, 2007 ▸; Domagała *et al.*, 2012 ▸; Nassour *et al.*, 2017 ▸), Invariom (Dittrich *et al.*, 2004 ▸, 2005 ▸, 2006 ▸, 2013 ▸), UBDB (Volkov *et al.*, 2007 ▸; Dominiak *et al.*, 2007 ▸; Jarzembska & Dominiak, 2012 ▸; Kumar *et al.*, 2019 ▸) and its successor MATTS (Jha *et al.*, 2022 ▸). The term transferable aspherical atom model (TAAM) was coined for the model they use. It has been shown that TAAM derived hydrogen atom parameters were more accurate than those obtained using IAM (*e.g.* Bąk *et al.*, 2011 ▸; Jha *et al.*, 2020 ▸).

HAR is based on atomic electron densities obtained by Hirshfeld partition of the electron density calculated for a system of interest using quantum chemical methods. Most often, the wavefunction calculations are performed for a non-periodic system consisting of an isolated molecule or a cluster of chemical units. Introduction of surrounding point multipoles representing the electrostatic potential in a crystal improves the accuracy of the resulting model (Jayatilaka & Dittrich, 2008 ▸; Capelli *et al.*, 2014 ▸; Chodkiewicz *et al.*, 2020 ▸). Recently, HAR based on calculation of periodic wavefunctions was tested (Ruth *et al.*, 2022 ▸) and it showed improvement over methods which neglected the crystal environment or treated it classically. TAAM neglects the environment and relies on the transferability of atomic electron densities. It also uses less a flexible electron density model than HAR (Jayatilaka & Dittrich, 2008 ▸; Koritsanszky *et al.*, 2011 ▸). As a result, HAR provides more accurate results than TAAM (Sanjuan-Szklarz *et al.*, 2020 ▸; Chodkiewicz *et al.*, 2020 ▸; Jha *et al.*, 2020 ▸). Yet the time needed for TAAM form factor calculations is usually much shorter than in the case of HAR (Jha *et al.*, 2023 ▸) and it is of the same order as the time taken by IAM. Two approaches have been developed for coping with relatively long computational times in the case of HAR. Fragmentation based methods (Bergmann *et al.*, 2020 ▸; Chodkiewicz *et al.*, 2022 ▸) use a fragmentation approach from quantum chemistry (Gordon *et al.*, 2012 ▸; Collins & Bettens, 2015 ▸; Raghavachari & Saha, 2015 ▸; Herbert, 2019 ▸) by dividing the system into fragments and performing wavefunction calculations for the smaller subsystems, which is faster than calculations for the whole, large system. These approaches, similar to TAAM, use the concept of transferability of electron densities, in this case from a fragment to the whole system. HAR-ELMO (Malaspina *et al.*, 2019 ▸) also relies on transferability. In this case, application of ELMOdb (Meyer & Genoni, 2018 ▸), which uses extremely localized molecular orbitals (ELMOs) (Meyer *et al.*, 2016 ▸) in wavefunction calculations, which, combined with HAR, allows for significantly faster calculations than original HAR. Similar to TAAM, it is restricted to a small number of atom types whereas the HAR-ELMO approach is restricted by the limited availability of precomputed molecular orbitals. An approach combining HAR and TAAM where the form factors for some atoms were calculated with HAR and others with TAAM was introduced (Jha *et al.*, 2023 ▸). Another way to combine ideas used in TAAM and HAR is to create a database of atomic densities – similar to the case of TAAM, and as in HAR – to use Hirshfeld partition of electron density to obtain the atomic densities. The Hirshfeld partition is applied to wavefunction based electron densities of molecules and ions selected for databank generation. Resulting atomic densities are stored in the database and can be later used by transferring them from the database to similar atoms in the structure under investigation. Such a databank was already constructed (Koritsanszky *et al.*, 2011 ▸) and the transferability of Hirshfeld partition based atomic densities was demonstrated, yet application to experimental results was discussed only very briefly. An application of such transferability was also studied by Chodkiewicz *et al.* (2022 ▸), where atomic electron densities of some atoms in the structure were described using the electron densities of other similar atoms from that structure. This approach gave results very similar to standard HAR when the densities were transferred between chemically similar atoms involved in some similar intermolecular interactions.

This work is a pilot study on the development of atomic density databanks obtained by the partition of electron densities derived from quantum mechanically calculated molecular/ionic wavefunctions. Hereafter, we will call this model the transferable Hirshfeld atom model (THAM). Two crucial aspects of refinements with such databanks were tested: accuracy and speed. Accuracy assessment for HAR-related methods is a complex task since HAR can be performed with various combinations of settings including the choice of quantum chemistry method and wavefunction used. Though these aspects were studied in many works (Capelli *et al.*, 2014 ▸; Fugel *et al.*, 2018 ▸; Chodkiewicz *et al.*, 2020 ▸; Wieduwilt *et al.*, 2020 ▸; Ruth *et al.*, 2022 ▸), no clear general protocol has been established for choosing the theory level. Therefore, in this work we construct three databanks using a different set of quantum chemical methods and basis sets to show various aspects of the method. To make things more complex, the Hirshfeld electron density partition can be replaced by other partitions (Chodkiewicz *et al.*, 2020 ▸) and there is no clear choice for the best method. Therefore, it is impossible to provide the final assessment of HAR as a method in general, since the method can only be tested for a particular combination of settings.

## 2. Theory

Atomic form factors in HAR are calculated via numerical integration of atomic electron densities which are evaluated at integration grid points around atoms. Form factors *f_a_* (**S**) of atom *a* are then calculated via the following summation:

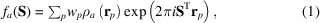

which runs over integration grid points, 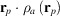 is the electron density of atom *a* and *w*_p_ is the integration weight. The integration grid is created by a combination of two integration grids: radial and angular. It contains points of type 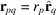 , where *r_p_* is a point in a radial integration grid (a scalar) and 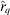 is a point in an angular integration grid (a normalized vector). The atomic electron densities for predefined atom types can be stored in a databank and reused for the calculation of atomic form factors. Here we assume that the electron densities are approximately transferable between atoms of the same atom type, *e.g.* between two hydrogen atoms of an alcohol hydroxyl group. Transfer of density from the databank to an atom in a structure involves reorientation of that density to match the local chemical environment. Similar to TAAM, a local coordinate system is defined for each atom type, *e.g.* for the hydrogen in an alcohol OH group one could choose a *Z* axis along the H—O bond, a *Y* axis lying at the H—O—C plane and perpendicular to *Z* and *X* axes in the direction of a vector product of the *Y* and *Z* directions. The atomic form factor *f_a_* (**S**) for the atom in the structure corresponds to *f_a_* (**M**_*a*_ **S**) calculated for the atomic density stored in the databank, where **M**_*a*_ is a matrix where the rows are given by unit local coordinate system vectors. Calculation of summation (1) is time consuming, since for each atom, each grid point and each reciprocal space vector **S** the expression 
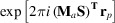
 has to be evaluated. For HAR, local coordinate systems do not appear and instead an 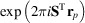 term is evaluated which is identical for atoms of the same chemical elements. Still, even in the case of HAR, the time needed for these calculations can be comparable to the time needed for wavefunction calculations when fragmentation based quantum chemistry methods are used (Chodkiewicz *et al.*, 2022 ▸). The problem can be mitigated by changing the representations of atomic electron densities. Expressing them in terms of spherical harmonics (Stewart, 1977 ▸; Koritsanszky & Volkov, 2004 ▸):

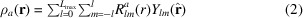

leads to the following expression for form factors:

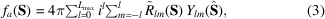

where 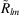 is a Hankel transform (also known as Fourier–Bessel transform) of radial function 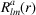 :

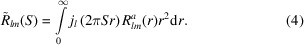

Evaluation of radial functions 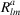 (see Appendix *A* [App appa]) is a relatively fast step, calculation of 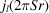 [which appears in equation (4)] is performed at points which are common to atoms of the same element and it also becomes unimportant for the calculation times of larger systems. Equation (4) can be computed by numerical integration and inserted in equation (3). If the integrations involve *N*_rad_ grid points, evaluation of equation (3) with *l* up to *L*_max_ requires the addition of 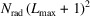 terms. In the case of HAR (for a version not involving the calculation of a periodic wavefunction), numerical integration calculations at this step involve *N*_rad_and *N*_ang_ terms where *N*_ang_ is the size of the angular grid, typically more than 300. As demonstrated later, *L*_max_ = 7 provides refinement results which are very similar to those obtained without the approximation [equation (2)]. This means that this part is performed roughly *N*_ang_/(7 + 1)^2^ ≃ 6–9 times faster than usual in the case of HAR. The multipole expansion (2) can be also used in HAR, yet it will lead to considerable speedup only in the cases when the calculation of the wavefunction is not the dominant factor, limiting speed (HAR incorporating fragment based quantum chemistry methods is a good candidate). Such a multipole expansion was also used in the earlier construction of the databank of atomic densities obtained with Hirshfeld partitions (Koritsanszky *et al.*, 2011 ▸). It can be treated as a technical procedure which speeds up calculations and preserves accuracy. THAM is a natural alternative to TAAM. The Hansen–Coppens multipole model, which is applied in TAAM, uses the following representation of atomic electron density:

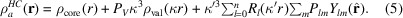

It is composed of a fixed term describing core electron density ρ_core_(*r*), a term describing the spherical part of valence density – 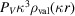 , which can be adjusted using two parameters: *P_V_* and κ, and a term expressed using the multipole expansion, where radial functions are expressed as single Slater functions independent of index *m* of the spherical harmonics. In comparison with THAM with numerical representations of radial functions, the flexibility of radial functions in the Hansen–Coppens model is much more limited. This is a desired property in the case of determination of experimental charge density since it allows the user to limit the number of refined parameters, but it is also a source of error in the description of the electron density (Koritsanszky *et al.*, 2011 ▸).

## 3. Multipole expansion of atomic electron density

**Figure 1:**
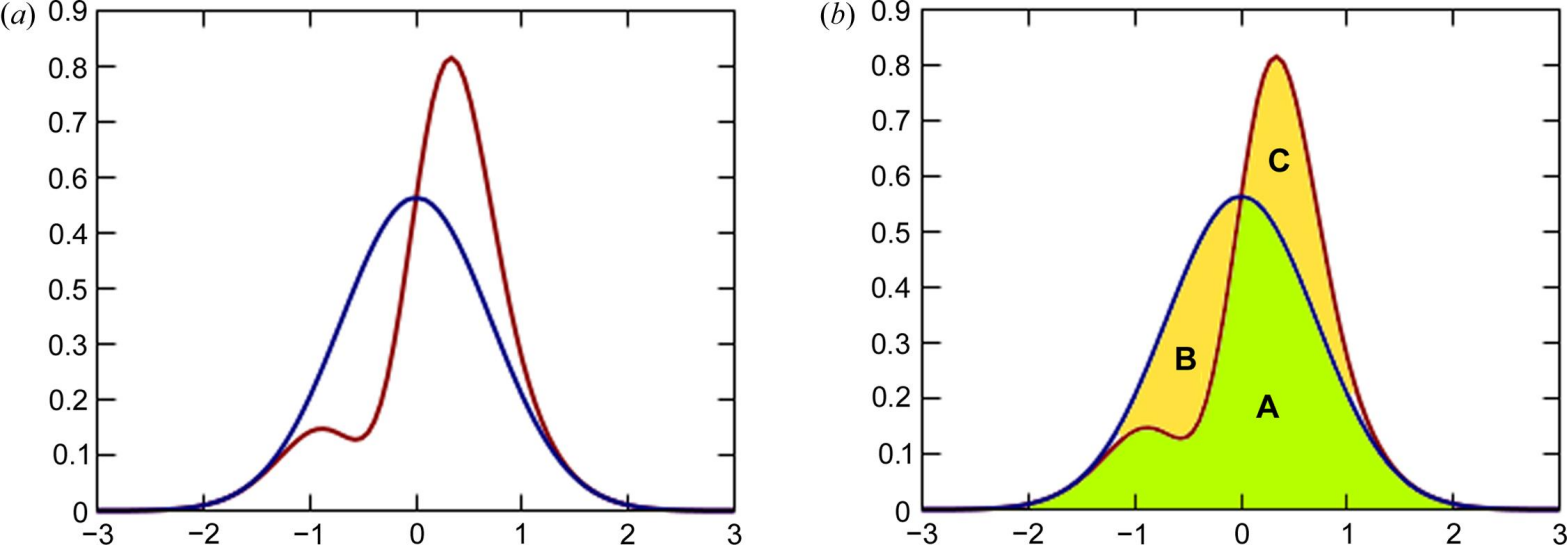
(*a*) The *S*_12_ index for the two probability distributions is 3.4, yet the percentage difference seems to be larger. (*b*) The overlapping (common) area is 0.78 (marked A). The overlapping coefficient of the PDFs corresponds to the area which would translate to a 22% difference.

**Figure 2:**
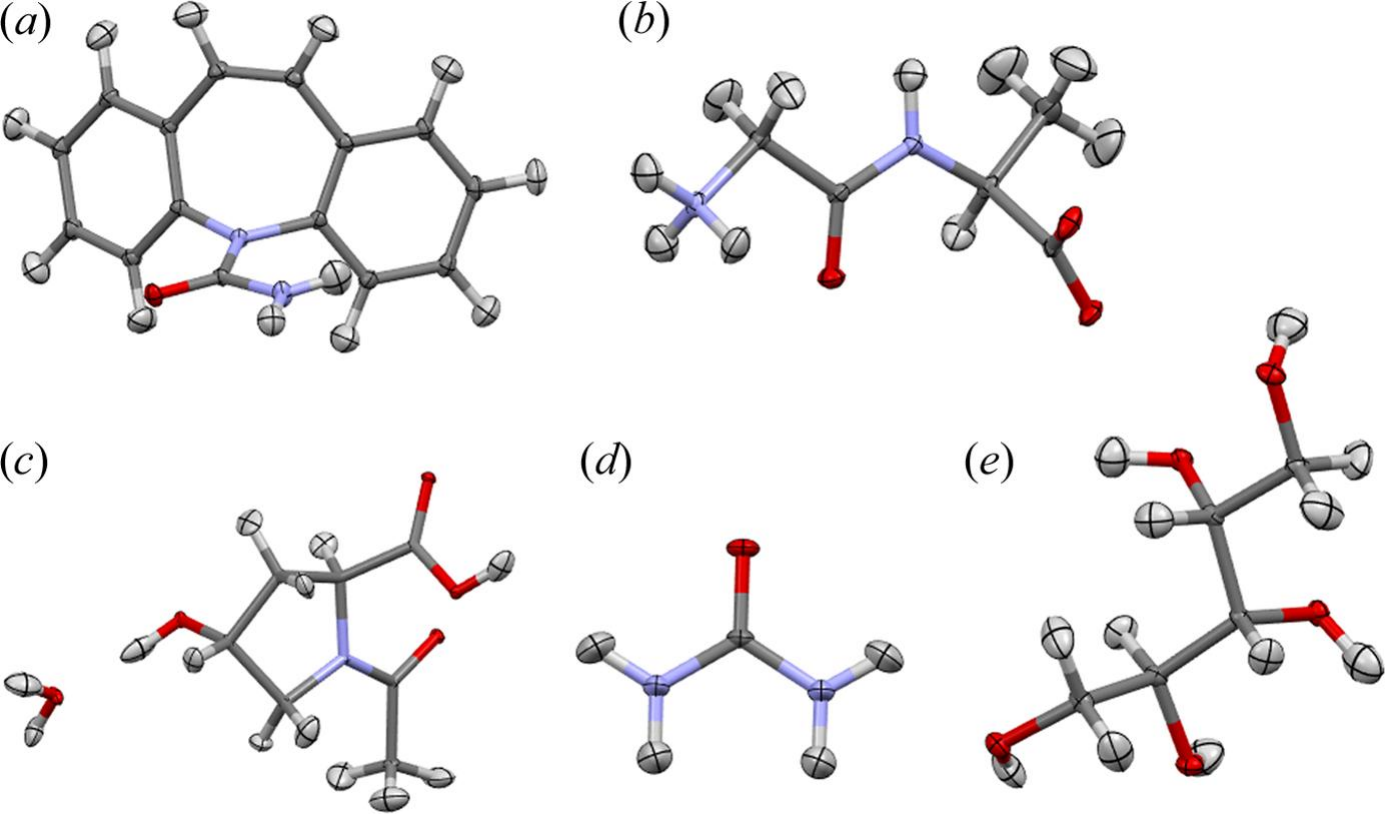
Test systems, structures refined with THAM bank build at the B3LYP/cc-pVTZ level of theory: (*a*) carbamazepine, (*b*) Gly-l-Ala, (*c*) NAC·H_2_O (*N*-acetyl-l-4-hy­droxy­proline monohydrate), (*d*) urea and (*e*) xylitol.

In order to assess the influence of multipole expansion of atomic electron densities [equation (2)] on refinement, some test HARs involving this expansion were performed for *L*_max_ up to 9 for the structures of carbamazepine, xylitol and urea. The results of the refinement were compared with structures obtained with the original HAR (*i.e.* without application of the multipole expansion). The comparison was focused on the structural parameters of the hydrogen atoms since they are the most sensitive to changes in the model. The lengths of covalent bonds to hydrogen and hydrogen atom atomic displacement parameters (ADPs) were compared. Bond lengths were compared with the average and maximal absolute difference between test and reference structures. For ADPs, the overlapping coefficient was applied (Inman & Bradley, 1989 ▸) which, in the case of one dimensional distributions, was simply the common area of two probability distribution functions (PDFs) on the plot (see Fig. 2). In the case of ADPs with the corresponding PDFs, *p*_1_(**u**) and *p*_2_(**u**) of the atomic displacement (**u**), the overlapping coefficient is defined as

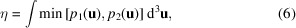

or alternatively with the help of the 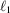 norm of the difference of PDFs as 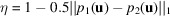 . In the rescaled form 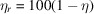 , it seems to correspond to the percentage difference between PDFs. Though such an interpretation has also been employed for the *S*_12_ APD similarity index introduced by Whitten & Spackman (2006 ▸), it sometimes seems to be counterintuitive (see Fig. 1 ▸). For example, when comparing the ADP tensor *U* and a two times larger tensor 2*U*, *S*_12_ is only 8.45 (see the supporting information for the derivation and comparison with η_r_).

Maximum values of the absolute difference in covalent *X*—H bond lengths and for hydrogen atoms η_r_ for the tested compounds are given in Table 1 ▸. The average values including values of the *S*_12_ similarity index and their plots are included in the supporting information. For *L*_max_ = 7, the maximum difference in the *X*—H bond length does not exceed 1 mÅ and the maximum η_r_ (percentage difference between PDFs corresponding to ADPs) is below 1. This indicates a very good agreement between structures refined with the original and approximate representations of atomic densities. Further calculations with the THAM model are performed with *L*_max_ = 7.

## 4. Creation of databanks

The databank employs atom-type definitions from the MATTS databank. Only atom types included in the test systems [xylitol, urea, carbamazepine, Gly-l-Ala,*N*-acetyl-l-4-hy­droxy­proline monohydrate (NAC·H_2_O) and crambin] are included in the constructed databank (52 atom types in total). For databank construction, a set of molecules/ions is chosen from a subset of structures included in the Cambridge Structural Database (CSD, Groom *et al.*, 2016 ▸) in such a way that, for each atom type, there are at least three molecules/ions containing atom(s) of that type. Then the wavefunction is calculated for each of the systems using the CSD geometric form with hydrogen atom positions extended to match reference neutron values from Allen & Bruno (2010 ▸). These calculations are performed with *ORCA* (version 5.0; Neese, 2012 ▸, 2022 ▸). Three different levels of theory were used, each corresponding to the creation of a separate databank: DFT with the B3LYP functional using a 6-31G(d,p) basis set (which matches the method used in the MATTS TAAM databank creation), the cc-pVTZ basis set and the Hartree–Fock with cc-pVTZ basis set. Using the calculated wavefunctions the atomic electron densities are calculated on integration grids oriented according to local coordinate systems of those atoms. Finally the atomic electron densities are averaged over all occurrences of each atom type and saved to text files.

The selection of the molecules/ions for wavefunction calculation is performed in the following way (see the supporting information for more information):

(1) Preliminary selection of structures from the CSD databank is performed.

(2) Further selection with the help of locally developed software. Structures with isotropic ADPs for non-hydrogen atoms are removed, only one member of each CSD Refcode family (*i.e.* structures with the same six-letter code) is retained – the one with the lowest maximal equivalent isotropic atomic displacement parameter (*U*_eq_). Then 40% of the structures with the highest values for their maximum *U*_eq_ in each structure are rejected.

(3) Final selection of molecules/ions. Each structure is divided into separate chemical units (molecules/ions) using the information available from the CSD. For each chemical unit, atom-type assignment is performed. Then the selection procedure is applied. It aims to choose a small number of chemical units, preferably of small size and diverse chemical composition. A total of 30 chemical units were selected, each containing between 3 and 39 atoms (17.5 on average).

## 5. Test of databanks

### 5.1. Test systems

Five existing datasets for small-structure systems (Fig. 2 ▸) were selected for testing the accuracy of the refinement with the THAM banks: xylitol – X-ray dataset published by Madsen *et al.* (2004 ▸) and neutron structure published by Madsen *et al.* (2003 ▸); urea – X-ray dataset published by Birkedal *et al.* (2004 ▸) and neutron measurement published by Swaminathan *et al.* (1984 ▸); carbamazepine, form III – both neutron and X-ray structures published by Sovago *et al.* (2016 ▸); Gly-l-Ala – both neutron and X-ray structures published by Capelli *et al.* (2014 ▸); and *N*-acetyl-l-4-hy­droxy­proline monohydrate (NAC·H_2_O) – both neutron and X-ray structures published by Lübben *et al.* (2014 ▸).

In addition, a high-resolution X-ray structure of the small protein crambin (Jelsch *et al.*, 2000 ▸) was used for the purpose of testing the execution time.

### 5.2. Execution time

Low computational cost is a key advantage of TAAM over HAR (Jha *et al.*, 2023 ▸). Such a property is also desirable in the case of THAM. We have tested two approaches: with and without multipole expansion of atomic densities. For comparison, the calculation time for a single HAR iteration is reported (at the B3LYP/cc-pVTZ level of theory, using default *ORCA5* convergence settings for wavefunction calculation). The results are included in Table 2 ▸ as the total execution time needed to run the program which generates a text file with the atomic form factors. For those of the tested systems which require longer computational times, calculations with multipole expansions were about 50 times faster than versions without this approximation. After partial parallelization of the problem [summation from equation (3) is parallelized], further speedup was achieved with a multicore CPU, and execution times dropped to less than 6 s, compared with ∼17 min for the version without multipole expansion and no parallelization.

We have also tested calculation times for a much larger system: the small protein, crambin. The disordered part of the protein was removed, leaving 642 atoms. Calculations were performed for data with resolution limited to 0.73 Å [the resolution used with the HAR-ELMO method (Malaspina *et al.*, 2019 ▸)]. Atomic form factors were calculated for 90 425 reflections. It took 68 s (using 12 CPU cores, all parallel calculations mentioned use one thread per core). For TAAM, calculations took about 8 s. For HAR-ELMO, the time for a single calculation of form factors was not reported, instead the total refinement time was given (6 days using a single CPU). For comparison, calculation of the wavefunction (one of the steps in the HAR procedure) for carbamazepine (30 atoms) at the B3LYP/cc-pVTZ level took 106 s (using 10 CPU cores) in the first HAR iteration and 57 s in the last HAR iteration (which is faster since the previous calculations are used as a starting point).

Although further speedup could be potentially achieved in the case of THAM, the current implementation already seems to allow for comfortable (comparable to IAM) use of the model in terms of execution time.

### 5.3. Testing THAM against HAR

One of the ways to test the effect of the transferability approximation utilized by THAM is to compare HAR- and THAM derived structures. The results of such comparison for both methods using the B3LYP/cc-pVTZ level of theory are presented in Table 3 ▸. The differences are similar in extent to those caused by switching from the cc-pVTZ basis set to cc-pVDZ HAR (Chodkiewicz *et al.*, 2022 ▸). Overall, they are usually smaller than the differences between HAR and neutron structures reported in the literature (*e.g.* Capelli *et al.*, 2014 ▸; Fugel *et al.*, 2018 ▸; Chodkiewicz *et al.*, 2020 ▸; Wieduwilt *et al.*, 2020 ▸; Ruth *et al.*, 2022 ▸).

### 5.4. Test against neutron data: comparison with other methods

Refinements with the following models were compared with the results from neutron measurements.

(1) Independent atom model (IAM), the most popular model; it neglects the asphericity of atomic electron densities.

(2) Transferable aspherical atom model (TAAM); the MATTS databank is used.

(3) THAM/B3LYP/6-31G(d,p) – calculated with the same theory level and atom type definitions as TAAM – chosen to perform a fair comparison with the TAAM bank.

(4) THAM/B3LYP/cc-pVTZ: TAAM calculated with the same functional (B3LYP) but about two times larger basis set (cc-pVTZ). Switching to an even larger (quadruple zeta) basis set in HAR does not seem to lead to a clear improvement in refinement statistics (*e.g.* Capelli *et al.*, 2014 ▸; Chodkiewicz *et al.*, 2020 ▸; Wieduwilt *et al.*, 2020 ▸).

(5) HAR(±) B3LYP/6-31G(d,p): HAR with the crystal environment represented via point multipoles calculated using the B3LYP/6-31G(d,p) theory level (*i.e.* theory level similar to that used in TAAM).

(6) HAR B3LYP/cc-pVTZ: HAR using the B3LYP/cc-pVTZ theory level, without representation of the crystallographic environment (*i.e.* the HAR version which is the most similar to THAM from point 4).

(7) HAR B3LYP/cc-pVTZ (±): HAR with the crystal environment represented via point multipoles – also calculated at the B3LYP/cc-pVTZ level. Representation of the crystal field is recommended for systems with hydrogen bonds as it results in more accurate structures (*e.g.* Capelli *et al.*, 2014 ▸; Chodkiewicz *et al.*, 2020 ▸, 2022 ▸). The computational cost of using point multipoles is usually relatively small (*e.g.* calculation of the wavefunction for crambin with and without the multipoles took 58.6 and 57 s respectively).

(8) THAM Hartree–Fock/cc-pVTZ: a version of the THAM bank generated using the Hartree–Fock method. HAR accuracy depends (among others) on the choice of quantum chemistry method and basis set. The same could be expected for THAM. This version of the THAM bank was included in the comparison to investigate this dependence.

(9) HAR Hartree–Fock/cc-pVTZ (±): HAR with the crystallographic environment represented via point multipoles, calculated at the Hartree–Fock/cc-pVTZ level.

Results for model 5 are presented in the supporting information (Table S5), these were added to provide a comparison of TAAM, THAM and HAR(±) that use the same level of theory [B3LYP/6-31G(d,p)].

#### 5.4.1. Figures of merit

**Figure 3:**
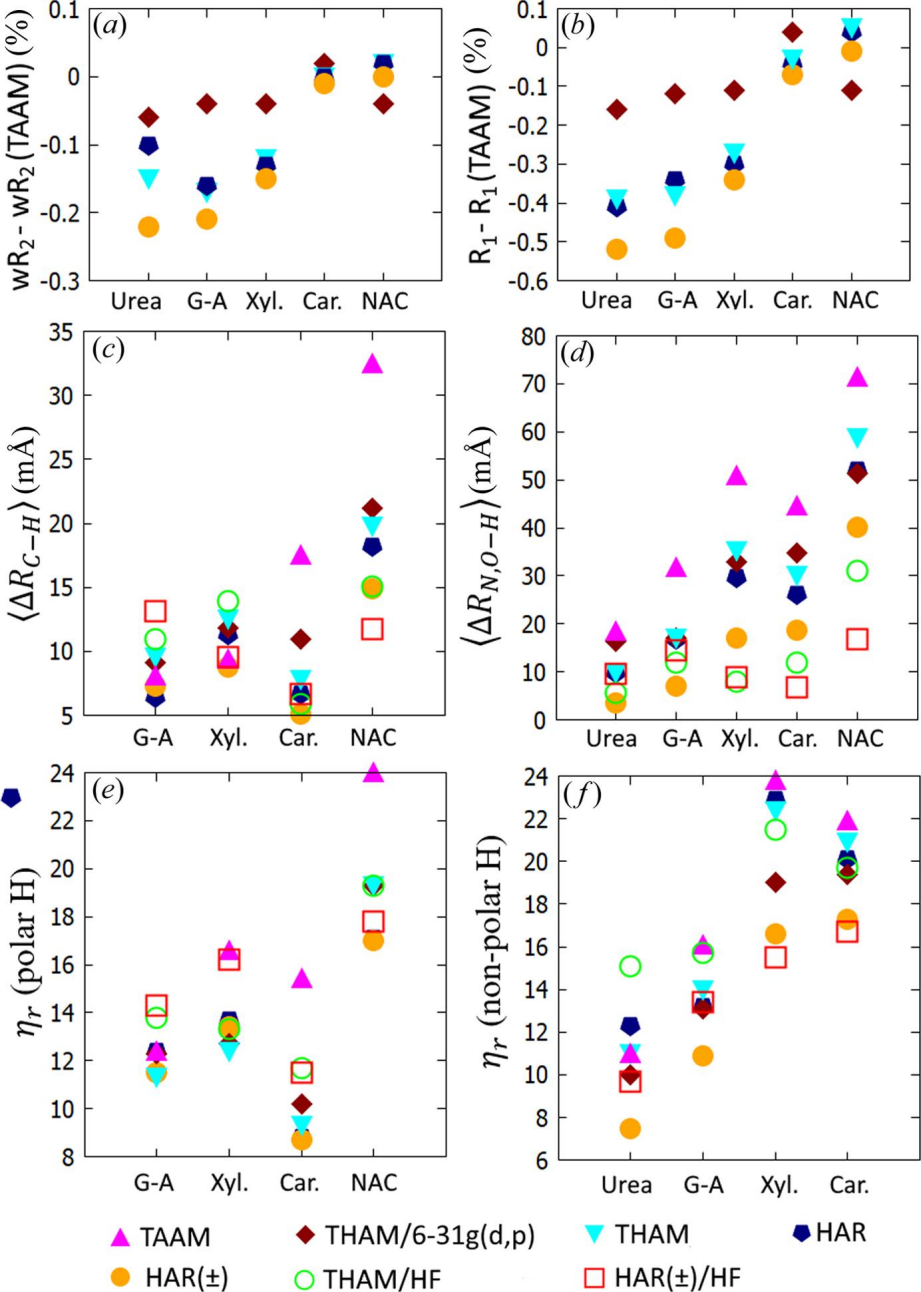
Comparison of (*a*) *wR*_2_ and (*b*) *R*_1_ agreement factors (as a difference between the factor and its value for TAAM), *X*—H bond lengths with reference neutron diffraction data in terms of average absolute difference (in mÅ) for (*c*) nonpolar (C—H) and (*d*) polar (N—H and O—H) bonds, hydrogen ADPs with reference neutron diffraction data in terms of the average rescaled overlapping coefficient η_r_ for (*e*) nonpolar and (*f*) polar hydrogen atoms. THAM and HAR models are based on B3LYP/cc-pVTZ unless specified otherwise, (±) represents HAR with the crystal environment represented via point multipoles. Agreement factors for Hartree–Fock based models and η_r_ for polar hydrogen atoms in NAC·H_2_O are omitted for clarity. Structure abbreviations: G-A – Gly-l-Ala, Xyl. – Xylitol, Car. – Carbamazepine, NAC – NAC·H_2_O.

It could be expected that the model accuracy increases in the following order (models using B3LYP): THAM bank created with (1) smaller [G-31G(d,p)] and (2) larger (cc-pVTZ) basis sets, then HAR (3) without and (4) with the effects of the crystal environment represented. It does not have to be strictly reproduced since various sources of inadequacy in modelling experimental data can interfere with each other and cause either addition or cancellation of errors. In terms of the *wR*_2_ and *R*_1_ agreement factor [Table 4 ▸ and Figs. 3 ▸(*a*) and 3 ▸(*b*)], this order clearly is not followed in the case of NAC·H_2_O, but for the other systems it is, with one exception: the order of THAM and HAR at the B3LYP/cc-pVTZ theory level as sometimes HAR gives larger *wR*_2_ or *R*_1_ than THAM. For methods based on Hartree–Fock, a method corresponding to lower *R*_1_ cannot be named, even though HAR incorporates crystal environmental representation in this case. For all of the cases with the exception of NAC·H_2_O, TAAM gives a larger *R*_1_ than THAM developed with the same method (B3LYP). These results suggest that THAM reproduces scattering factors more accurately than TAAM and with similar accuracy to the version of HAR that neglects the crystal environment.

#### 5.4.2. Comparison of *X*—H bond lengths

Improvement of the structural parameters for hydrogen atoms is one of main advantages of aspherical models of atomic densities over spherical ones. Though IAM significantly underestimates the lengths of covalent bonds to hydrogen atoms by 10% or more, HAR and TAAM give much more accurate bond lengths – usually differing from the values obtained with neutron diffraction by less than 0.03 Å (sometimes even below 0.01 Å). The O—H and N—H bond lengths are more challenging to accurately reproduce with HAR than the C—H bond lengths; therefore, statistics for polar and nonpolar *X*—H bonds are presented separately. The comparison of the X-ray and neutron derived values of the bond lengths in terms of the average absolute difference is presented in Table 5 ▸ and in Figs. 3 ▸(*c*) and 3 ▸(*d*).

#### 5.4.3. B3LYP based methods

The test systems were chosen to cover cases with a diverse accuracy of HAR derived *X*—H (element-hydrogen) bond lengths. Although the C—H bond lengths for all methods based on the larger of the basis sets used (cc-pVTZ) give a discrepancy below 20 mÅ on average, the differences are considerably larger for a number of systems involving polar bonds – 30 mÅ and above for carbamazepine, xylitol and NAC·H_2_O. The IAM results are clearly inferior to those obtained with the aspherical atom model. In further discussion we focus on aspherical atom models only. The improvement of the C—H bond lengths is not as clear, nor is the improvement in the *R* factors, when switching to theoretically more accurate methods. The TAAM results were sometimes as good as HAR results, yet discrepancies for HAR when including the crystal environment treatment are always lower than for TAAM. TAAM is sometimes slightly better than THAM, but THAM is sometimes significantly better than TAAM. The improvement of *X*—H bond lengths when using more advanced methods is much more visible in the case of polar *X*—H bonds. The expected order of accuracy is almost always followed, with the exception of THAM based on cc-pVTZ being sometimes more accurate than HAR (similarly as in the case of the *R*_1_ statistic). An advantage of the Hirshfeld partition based methods over TAAM is quite clear in the case of polar *X*—H bonds.

#### 5.4.4. Hartree–Fock based method and its comparison with the B3LYP based method

In the case of HAR, it was observed that the use of DFT methods usually gives shorter bonds to the polar hydrogen atoms than the Hartree–Fock method (Capelli *et al.*, 2014 ▸; Chodkiewicz *et al.*, 2020 ▸; Wieduwilt *et al.*, 2020 ▸; Landeros-Rivera *et al.*, 2023 ▸). This difference was linked to an amount of Hartree–Fock exchange in the DFT functionals (Landeros-Rivera *et al.*, 2023 ▸). The O—H and N—H bond lengths, resulting from DFT based HAR, are, on average, shorter than those from neutron diffraction (Woińska *et al.*, 2016 ▸). Similar behaviour could be also expected in the case of THAM. The discrepancies in HAR derived bond lengths might appear to be quite large for some of the tested systems, but this is not a property of HAR in general, but rather HAR paired with a particular quantum chemical method. A THAM databank generated using the Hartree–Fock method gives more accurate results than HAR with B3LYP for cases where the X-ray neutron structure discrepancies were the largest (*i.e.* carbamazepine, NAC·H_2_O and xylitol). For those structures, the polar *X*—H bonds are already too short in the case of HAR performed with Hartree–Fock and the use of DFT with the B3LYP functional makes them even shorter, leading to larger discrepancies (see Table S3 of the average differences in *X*—H bond lengths). Though THAM developed with the Hartree–Fock method gives clearly better bond lengths than THAM developed with B3LYP, it also almost always gives visibly larger *R* factors (with the exception of the peculiar case of NAC·H_2_O). Unlike in the case of the B3LYP results, the THAM results are not clearly inferior to those obtained using HAR with crystal environment representation [HAR(±)] when Hartree–Fock is used. *X*—H bond lengths (Fig. S3) for polar hydrogen atoms obtained with HAR(±) are on average longer than in the case the corresponding THAM results, for both B3LYP and Hartree–Fock based methods. In the case of the THAM databank obtained with the B3LYP functional, the bonds are, on average, too short and their elongation when HAR(±) is applied leads to more accurate bond lengths. In the case of Hartree–Fock based THAM and HAR(±), the bond lengths are systematically longer than in the case of the B3LYP based methods. As a result, further elongation of THAM derived bonds with HAR do not lead, in general, to more accurate bond lengths in this case.

#### 5.4.5. Comparison of hydrogen atomic displacement parameters

Atomic displacement parameters are compared using rescaled overlapping coefficients [Table 6 ▸ and Figs. 3 ▸(*e*) and 3 ▸(*f*)], the values of the *S*_12_ similarity index are provided in the supporting information. Note that, in the case of carbon-bonded hydrogen atoms, all Hirshfeld partition based methods using the B3LYP functional showed similar performances, usually better than TAAM. This is also the case for polar hydrogen atoms. In this case, a clear advantage of representing the crystal environment can be noted. THAM and HAR with no such representation perform similarly. When comparing THAM banks based on the B3LYP functional and Hartree–Fock, it is not possible to choose the superior one. The THAM results can be summarized similarly to the case of comparison of bond lengths – it has a similar performance to HAR without representation of the crystal environment, usually worse than HAR with such a representation and better than TAAM.

## 6. Conclusions

With the advent of aspherical atom models in X-ray refinement, it becomes clear than they lead to much more accurate structural parameters than spherical model of atomic electron density, especially in the case of hydrogen atom parameters. In fact, a significant improvement can be obtained with TAAM at a computational cost that is negligible for small-molecule structures while further improvement can be gained using HAR at the cost of significantly increasing computational time. In this paper, we test the model which combines ideas utilized in TAAM and HAR by constructing databases of transferable atomic electron densities obtained by applying Hirshfeld partition to molecular electron densities. The resulting model is abbreviated to THAM. Similar ideas were utilized by Koritsanszky *et al.* (2011 ▸) in construction of the database of atomic electron densities. Here, we present the first detailed analysis of performance of this kind of databank in terms of accuracy of structural parameters of hydrogen atoms as well as computational performance.

The databank stores the atomic electron densities as the values at the point of integration grid used in HAR. Although for calculation of atomic form factors it is possible to directly use such representation, the resulting procedure is quite slow. Multipole expansion of atomic electron densities derived from Hirshfeld partition (Koritsanszky & Volkov, 2004 ▸) is used instead. It has been shown that such an expansion almost exactly reproduces reference results in terms of structural parameters of refined structures when spherical harmonics up to the seventh order are included. This procedure also allows for much faster calculations of atomic form factors. For the small protein crambin (642 atoms after the removal of disordered sections), it took only 68 s (using 12 CPU cores) and a few seconds for ‘small’ molecules – quick enough to be used as a replacement of the traditional IAM model without sacrificing speed.

The THAM databank and the TAAM bank MATTS have a common atom-typing mechanism; therefore, using also the same atom-type definitions and underlying quantum chemical theory level (B3LYP/cc-pVTZ) allows construction of the THAM databank which can be used to compare performances of THAM and TAAM. In addition, the THAM databank based on a larger basis set (cc-pVTZ) was created to explore the effect of the basis set. A third bank was constructed with the Hartree–Fock method and cc-pVTZ basis set to examine the influence of the quantum chemical method on THAM performance. A test refinement using TAAM, THAM and HAR lead to the following observations.

(i) Comparison of *R*_1_ form factors suggests that THAM reproduces scattering factors more accurately than TAAM and with similar accuracy to the version of HAR which neglects the crystal environment [indicated as HAR(−) and the version which does not neglect HAR(+)]. Use of a larger basis set in THAM construction lowers the *R* factors.

(ii) Comparison of the element hydrogen (*X*—H) covalent bond lengths with reference values from neutron experiments shows that all aspherical models tested led to C—H bond lengths of similar accuracy, yet HAR(+) performed slightly better than TAAM. The situation was different in the case of polar *X*—H bonds such as N—H and O—H. In this case, THAM was clearly more accurate than TAAM, HAR(−) was slightly more accurate than THAM and HAR(+) was clearly more accurate than HAR(−).

(iii) THAM and HAR reproduced nonpolar hydrogen ADPs with similar accuracy, usually better than TAAM, similarly to polar hydrogen atoms but here HAR(+) was clearly more accurate than the other methods.

(iv) In the case of HAR and THAM results for the model using DFT with the B3LYP functional and using Hartree–Fock were included. Use of Hartree–Fock led to longer polar *X*—H bonds which, for the structures tested, usually led to more accurate bond lengths. On the other hand, it was also associated with a higher *R* factor. None of the quantum chemical methods had a clear advantage in terms of ADP accuracy.

(v) In the case of B3LYP based methods, HAR(+) was more accurate than THAM in terms of *R* factors, *X*—H bond lengths and hydrogen atom ADPs. But, in cases of Hartree–Fock based methods, it was more accurate only in terms of the ADPs.

(vi) Although use of the larger basis set with THAM led to lower *R* factors, it did not translate to clearly better structural parameters for hydrogen atoms.

THAM has been shown to be more accurate than TAAM, almost as accurate as the version of HAR which neglects the crystal environment and less accurate than the version which takes it into account. Besides being a good replacement for IAM and an alternative for TAAM, it can be a convenient replacement for HAR when use of the most accurate possible method is not required. THAM is also a good first step before HAR since it brings the geometry of the investigated structure close to the HAR results which is important for reducing the number of HAR iterations (steps containing wavefunction calculation). Since THAM reproduced nonpolar hydrogen atom parameters with similar accuracy to HAR, it is potentially a good candidate for use in a hybrid approach (Jha *et al.*, 2023 ▸), where part of the form factors could be calculated with one method and part with the other (*e.g.* THAM could be used for the part containing nonpolar hydrogen atoms and HAR for the part with polar ones).

The tested THAM databanks contain 52 atom types (each) and were constructed for preliminary testing purposes. Before releasing a THAM databank for general use, we plan to explore a few more aspects of such databanks which include the choice of optimal quantum chemistry theory level and electron density partitions, definitions of atom types which work for a wide range of chemical systems, and further speed optimization of the method.

## 7. Methods

For HAR, a locally modified version of *Olex2* (Dolomanov *et al.*, 2009 ▸) was used in the refinements incorporating *discamb2tsc* (Chodkiewicz *et al.*, 2020 ▸) based on the *DiSCaMB* library (Chodkiewicz *et al.*, 2018 ▸) which generates files with atomic form factors in tsc format (Kleemiss *et al.*, 2021 ▸; Midgley *et al.*, 2019 ▸). Such files are then imported into *Olex2* and used in the refinement. https://dictionary.iucr.org/Refinement Details of the implementation are given by Chodkiewicz *et al.* (2022 ▸), the current implementation uses a different radial integration grid (Mura & Knowles, 1996 ▸) for calculation of the atomic form factors.

A rescaled overlapping index η_r_ is calculated with a program developed using the *DiSCaMB* library. The integral to be evaluated is separated into a radial and an angular part, numerical integration is used for the latter using a 5810 point grid (Lebedev & Laikov, 1999 ▸). The radial part is expressed in terms of an erf function and no numerical integration is necessary.

The chemical unit selection algorithm used in databank creation uses the VF2 graph isomorphism algorithm (Cordella *et al.*, 2004 ▸) as implemented in the *VFlib* library (https://mivia.unisa.it/vflib/).

## Supplementary Material

Zipped file containing all cifs. DOI: 10.1107/S2052252524001507/lt5065sup1.zip

Supporting figures and tables. DOI: 10.1107/S2052252524001507/lt5065sup2.pdf

## Figures and Tables

**Table 1:** Comparison of structures obtained from HAR refinement using atomic electron density represented (i) with numerical values on the integration grid (standard approach) (ii) with multipole expansion of the densities (up to *L*_max_ order of spherical harmonics). Reported maximum difference in length of the covalent bond to atoms (max|Δ*R*_*X*—H_|) and maximum rescaled overlapping coefficient (max η_r_) for ADP (see text).

*L*_max_	max|Δ*R*_*X*—H_| (mÅ)			max η_r_		
	Xylitol	Carbamazepine	Urea	Xylitol	Carbamazepine	Urea
3	8.59	6.43	6.10	7.99	4.48	6.26
4	3.90	3.16	6.08	4.03	2.21	2.93
5	2.89	2.24	3.06	2.71	1.71	2.83
6	1.61	1.25	0.99	1.08	0.72	0.65
7	0.95	0.64	0.47	0.96	0.42	0.42
8	0.78	0.43	0.28	0.80	0.52	0.25
9	0.62	0.25	0.16	0.54	0.28	0.18

**Table 2:** Elapsed real time (wall time) of execution of the program which calculates atomic form factors using the THAM model with and without multipole expansion of atomic electron densities, and also the time for the first iteration of HAR (B3LYP/cc-pVTZ, wavefunction calculation/total time) ‘Serial’ represents serial execution (*i.e.* using only one thread on one CPU).

Structure	Calculation time (s)			
	No multipole expansion (serial)	Multipole expansion (serial)	Multipole expansion (parallel with 10 CPU cores)	HAR (parallel with 10 CPU cores)wavefunction/all
Carbamazepine	1048	24	5.5	106/122
Gly-L-Ala	107	4.0	1.3	38/41
NAC·H_2_O	465	13	3.4	47/57
Urea	37	3.8	0.8	6/9
Xylitol	488	9.7	2.7	35/40

**Table 3:** Comparison of THAM refinement derived hydrogen atom parameters with results from HAR in terms of (i) the average absolute difference of covalent *X*—H bond lengths (in mÅ), (ii) differences in hydrogen atom ADPs measured with an *S*_12_ similarity index and (iii) rescaled overlapping index η_r_ Hydrogen atoms bonded to carbon are analysed separately from those bonded to oxygen and nitro­gen.

	〈|Δ*R*_*X*—H_|〉		*S*_12_		η_r_	
Hydrogen bonded to	C	O, N	C	O, N	C	O, N
Carbamazepine	2.3	3.9	0.21	0.50	3.6	7.0
Gly-L-Ala	6.8	3.9	0.48	0.82	6.5	8.3
NAC·H_2_O	4.4	6.6	0.57	1.33	6.6	10.8
Urea	–	5.8	–	0.49	–	6.8
Xylitol	6.5	5.6	0.42	0.28	6.1	4.8

**Table 4:** Comparison of *R*_1_ and *wR*_2_ agreement factors HAR (±) represents HAR with the crystal environment represented via point multipoles and HAR for the version without such representation.

		B3LYP method					Hartree–Fock method	
		G-31G(d,p) basis set		cc-pVTZ basis set				
Model	IAM	TAAM	THAM	THAM	HAR	HAR (±)	THAM	HAR (±)
*R*_1_								
Carbamazepine	4.07	2.62	2.64	2.62	2.62	2.61	2.75	2.78
Gly-L-Ala	2.40	1.56	1.52	1.39	1.40	1.35	1.58	1.59
NAC·H_2_O	3.18	2.46	2.42	2.48	2.48	2.46	2.43	2.42
Urea	2.45	1.56	1.50	1.41	1.46	1.34	1.54	1.47
Xylitol	2.39	1.71	1.67	1.59	1.58	1.56	1.65	1.65

*wR*_2_								
Carbamazepine	12.53	6.61	6.65	6.58	6.57	6.54	7.15	7.14
Gly-L-Ala	6.68	3.16	3.04	2.78	2.82	2.67	3.17	3.18
NAC·H_2_O	7.67	4.91	4.80	4.96	4.95	4.90	4.91	4.90
Urea	6.38	4.06	3.90	3.67	3.65	3.54	3.94	3.92
Xylitol	5.64	3.18	3.07	2.91	2.88	2.84	3.05	3.03

**Table 5:** Comparison of aspherical atom model derived *X*—H bond lengths with reference neutron diffraction data in terms of absolute difference (in mÅ) HAR (±) represents HAR with the crystal environment represented via point multipoles and HAR for the version without such representation.

		B3LYP method					Hartree–Fock method	
		G-31G(d,p) basis set		cc-pVTZ basis set				
Model	IAM	TAAM	THAM	THAM	HAR	HAR (±)	THAM	HAR (±)
C—H								
Carbamazepine	90	17.5	11	7.8	6.7	5.1	5.9	6.7
Gly-L-Ala	118	8.1	9.1	9.6	6.4	7.3	11.0	13.1
NAC·H_2_O	134	32.5	21.2	19.8	18.2	14.9	15.1	11.7
Xylitol	130	9.4	11.8	12.5	11.3	8.8	13.9	9.6

O—H, N—H								
Carbamazepine	157	44.5	34.8	30	26.1	18.6	12.0	6.7
Gly-L-Ala	143	31.7	17	17	16.9	7	11.9	14.5
NAC·H_2_O	138	71.3	51.2	58.8	52.1	40.1	31.0	16.7
Urea	98	18.5	16.3	9.9	9.9	3.6	5.7	9.6
Xylitol	124	50.9	33	35.2	29.6	17	7.9	9.0

**Table 6:** Comparison of hydrogen atom ADPs obtained with aspherical atom model X-ray refinements in terms of rescaled overlapping index η_r_, neutron diffraction experiments are used as a reference HAR (±) represents HAR with the crystal environment represented via point multipoles and HAR for the version without such representation.

	B3LYP method					Hartree–Fock method	
	G-31G(d,p) basis set		cc-pVTZ basis set				
	TAAM	THAM	THAM	HAR	HAR	THAM	HAR
Hydrogen bonded to C							
Carbamazepine	15.4	10.2	9.3	8.8	8.7	11.7	11.5
Gly-L-Ala	12.4	12.3	11.3	12.4	11.5	13.8	14.3
NAC·H_2_O	24.0	19.3	19.3	17.1	17.0	19.3	17.8
Xylitol	16.6	12.7	12.4	13.7	13.4	13.3	16.2

Hydrogen bonded to O or N							
Carbamazepine	21.9	19.4	20.9	20.1	17.3	19.71	16.7
Gly-L-Ala	16.1	13.1	14	13.2	10.9	15.7	13.4
NAC·H_2_O	36.1	29.0	31.1	28.9	23.7	29.9	25.9
Urea	11.0	10.0	11.0	12.3	7.5	15.1	9.7
Xylitol	23.8	19.0	22.4	22.9	16.6	21.5	15.5

## Appendix A Calculations of radial functions in multipole expansion of atomic electron densities

Radial functions  for atomic densities can be obtained by projection of the densities onto spherical harmonics:

They are calculated using numerical integration over a sphere:

Atomic electron densities {ρ_*a*_} are stored in the databank as numerical values calculated at points on the numerical integration grid suitable for calculation of atomic form factors. The grid is a composition of two grids: radial by Mura & Knowles (1996 ▸) and angular by Lebedev & Laikov (1999 ▸). The resulting grid contains points of type  , where *r_p_* is a point on a radial integration grid (a scalar) and  on an angular integration grid (a normalized vector).

In principle, atomic electron densities could be stored in a databank in the form of the radial functions  and adjusted for the local coordinate system when transferred to the structure of interest. However, at present, the less compact form is used with values for the whole integration grid stored. A value for the atomic electron density for a grid point with the coordinates [*a*, *b*, *c*] corresponds to the density at the point  in the structure to which the density is transferred, where  and  are unit vectors along the local coordinate system axes for the atom of interest in this structure, *i.e.*  , where  is an orthonormal matrix in which rows are given by  and  . An alternative set of angular grid points can be generated by multiplying the original grid points with any orthogonal matrix. Application of the  matrix for this purpose allows use of the values stored in the databank to calculate the radial functions:

Our implementation uses the right-hand sum in the above expression for this purpose.
