# Supplementary material for: Transferable Hirshfeld atom model for rapid evaluation of aspherical atomic form factors
Source: IUCrJ. 2024 Mar 1;11(Pt 2):249–59. doi: 10.1107/S2052252524001507 (PMC10916294; doi:10.1107/S2052252524001507)
Supplement: Supplementary file 2 [file m-11-00249-sup2.pdf]

IUCrJ

Volume 11 (2024)

Supporting information for article:

Transferable Hirshfeld atom model for rapid evaluation of aspherical atomic form factors

Michał Chodkiewicz, Leonid Patrikeev, Sylwia Pawlędzio and Krzysztof Woźniak

S1. Some properties of S_{12} similarity index

The S_{12} similarity index is defined as: $S_{12} = 100(1 - R_{12})$, where R_{12} is defined as follows:

$$R_{12} = \int \sqrt{p_1(\boldsymbol{u})p_2(\boldsymbol{u})}d^3\boldsymbol{u}$$

where $p(\boldsymbol{u})$ is probability density function (pdf) for finding atom displaced by vector $\boldsymbol{u}$ from the equilibrium. In fact R_{12} is equal to the correlation coefficient (Merrit, 1999), which is a popular measure used macromolecular crystallography, the equality can checked by comparing the exact expressions given in the original publications. R_{12} is known in statistics as the Bhattacharyya distance (Bhattacharyya 1943, 1946). The $p(\boldsymbol{u})$ pdf is given by:

$$p(\boldsymbol{u}) = \frac{1}{\sqrt{8\pi^3 \det U}} e^{-\boldsymbol{u}^T U^{-1}\boldsymbol{u}/2}$$

Where the matrix U is a mean-square displacement tensor which components are known as anisotropic atomic displacement parameters. Matrices U and U^{-1} has common eigenvectors. In coordinate systems which axis are oriented along eigenvectors of U the tensor becomes diagonal and the pdf becomes a product of three univariate Gaussian functions, each of the following form:

$$g(x,a) = \left(\frac{a}{\pi}\right)^{1/2} e^{ax^2}$$

The R_{12} integral becomes a product of three single variable integrals, each of the form:

$$R_{12}(a,b) = \int_{-\infty}^{\infty} \left(g(x,a)g(x,b)\right)^{1/2} dx = \left(\frac{4ab}{(a+b)^2}\right)^{1/4}$$

If a tensor U is compared with its *n*-times larger version nU, then each of the factors becomes:

$$R_{12}(a,na) = \left(\frac{4n}{(1+n)^2}\right)^{1/4} = R_{12}(n)$$

and as a consequence enlarging tensor U *n*-times and comparing with the original one would give:

$$S_{12}(n) = \left(1 - R_{12}^3(n)\right) * 100 = \left(1 - \left(\frac{4n}{(1+n)^2}\right)^{\frac{3}{4}}\right) * 100$$

which for *n*=2 (or ½) is about 8.455. I.e. when comparing tensor U with $2U$ the S_{12} similarity index takes such value. Expanding S_{12} into Taylor series around *n*=1 gives:

$S_{12}(n) = \frac{3}{16}(n-1)^2 - \frac{3}{16}(n-1)^3 + ..$

It shows that $S_{12}(n)$ behaves like a quadratic function of n around $n = 1$, i.e. it grows slowly in that region, for example enlarging ADPs by 5% gives S_{12} value about only 0.045 “percent” when compared with the original one.

Bhattacharyya, A. (1943) *Bull. Calcutta Math. Soc.* **55**, 99-110.
Bhattacharyya, A. (1946) *Indian J. Stat.* **7**, 401–406
Merritt, E. A. (1999). *Acta Cryst.* **D55**, 1997–2004.

S2. Example values of S_{12} and η_r

A comparison of values of similarity index S_{12} and rescaled overlapping index η_r is presented in Fig. S1. Xylitol and ice VI are used as test structures, neutron experiment structure of xylitol is taken from Madsen, A. Ø., Mason, S. & Larsen, S. (2003). Acta Cryst. B59, 653–663 and ice VI from Kuhs, W., Ahsbahs, H., Londono, D. & Finney, J. (1989). Physica B, 156–157, 684–687. The HAR structure for ice VI was taken from Chodkiewicz, M. L., Gajda, R., Lavina, B., Tkachev, S., Prakapenka, V. B., Dera, P. & Woźniak, K. (2022). IUCrJ, 9, 573-579 and for xylitol HAR was performed with the B3LYP functional and cc-pVTZ basis set with surrounding multipoles (up to 15 Å and up to quadrupoles, not exactly the same refinement as in the main body of the paper).

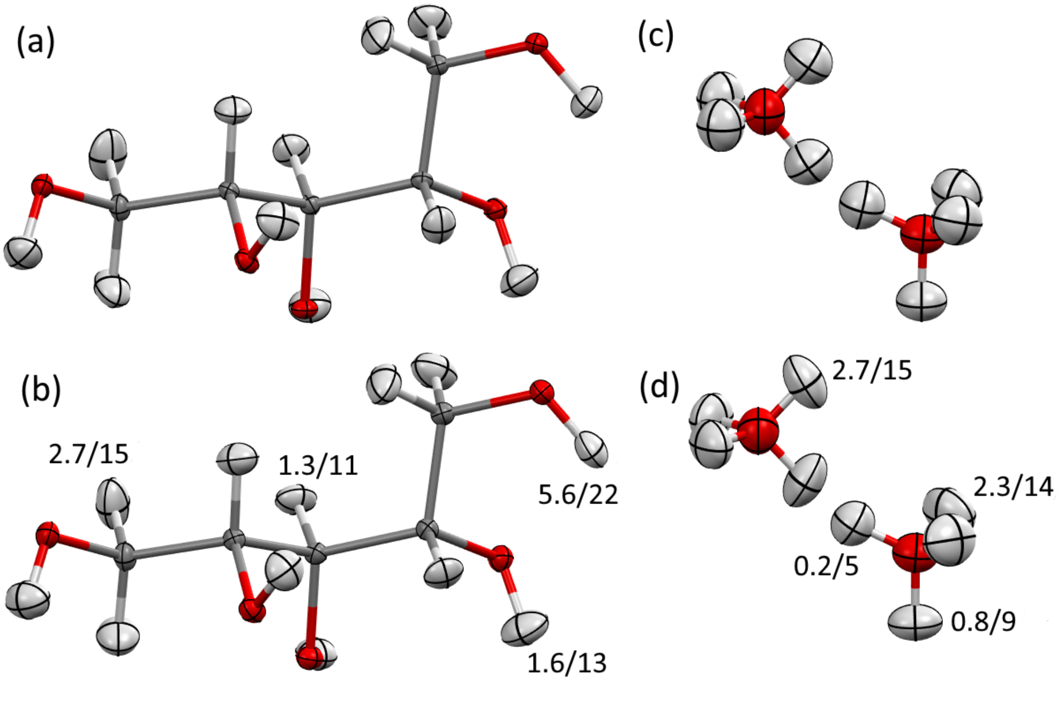

Figure S1 Comparison of ADPs similarity indices S_{12}/η_r for the xylitol structure (a) from neutron measurement and (b) from HAR and for ice VI (c) from neutron measurement and (d) from HAR.

S3. Accuracy of multipole expansion of atomic electron density in HAR

Table S1 Comparison of structures obtained from HAR refinement using atomic electron density represented (1) with numerical values on integration grid (standard approach) (2) with multipole expansion of the densities (up to L_{max} order of spherical harmonics). Reported average difference in length of covalent bond to atom ($\langle|\Delta R_{X-H}|\rangle$), and average rescaled overlapping coefficient ($\langle\eta_r\rangle$) for hydrogen atom ADPs.

L_{max}	$\langle\lvert\Delta R_{X-H}\rvert\rangle$ $(m\text{Å})$			$\langle\eta_r\rangle$		
	xylitol	carbamazepine	urea	xylitol	carbamazepine	urea
3	4.46	3.12	4.03	6.61	2.39	4.32
4	1.91	1.55	4.81	2.44	1.24	2.76
5	1.36	1.45	2.50	1.69	0.89	1.95
6	0.80	0.61	0.53	0.65	0.46	0.47
7	0.48	0.41	0.39	0.46	0.29	0.40
8	0.34	0.31	0.25	0.42	0.35	0.25
9	0.29	0.15	0.13	0.31	0.19	0.17

Table S2 Comparison of structures obtained from HAR refinement using atomic electron density represented (1) with numerical values on integration grid (standard approach) (2) with multipole expansion of the densities (up to L_{max} order of spherical harmonics). Reported average ($\langle S_{12}\rangle$) and maximum ($\max S_{12}$) ADPs similarity index S_{12}.

L_{max}	$\langle S_{12}\rangle$			$\max S_{12}$		
	xylitol	carbamazepine	urea	xylitol	carbamazepine	urea
3	0.4376	0.0658	0.2162	0.6189	0.1960	0.3816
4	0.0697	0.0180	0.0747	0.1725	0.0460	0.0850
5	0.0349	0.0096	0.0508	0.0808	0.0293	0.0895
6	0.0049	0.0025	0.0027	0.0128	0.0050	0.0045
7	0.0027	0.0010	0.0018	0.0103	0.0017	0.0019
8	0.0021	0.0014	0.0007	0.0072	0.0031	0.0008
9	0.0012	0.0004	0.0003	0.0032	0.0009	0.0004

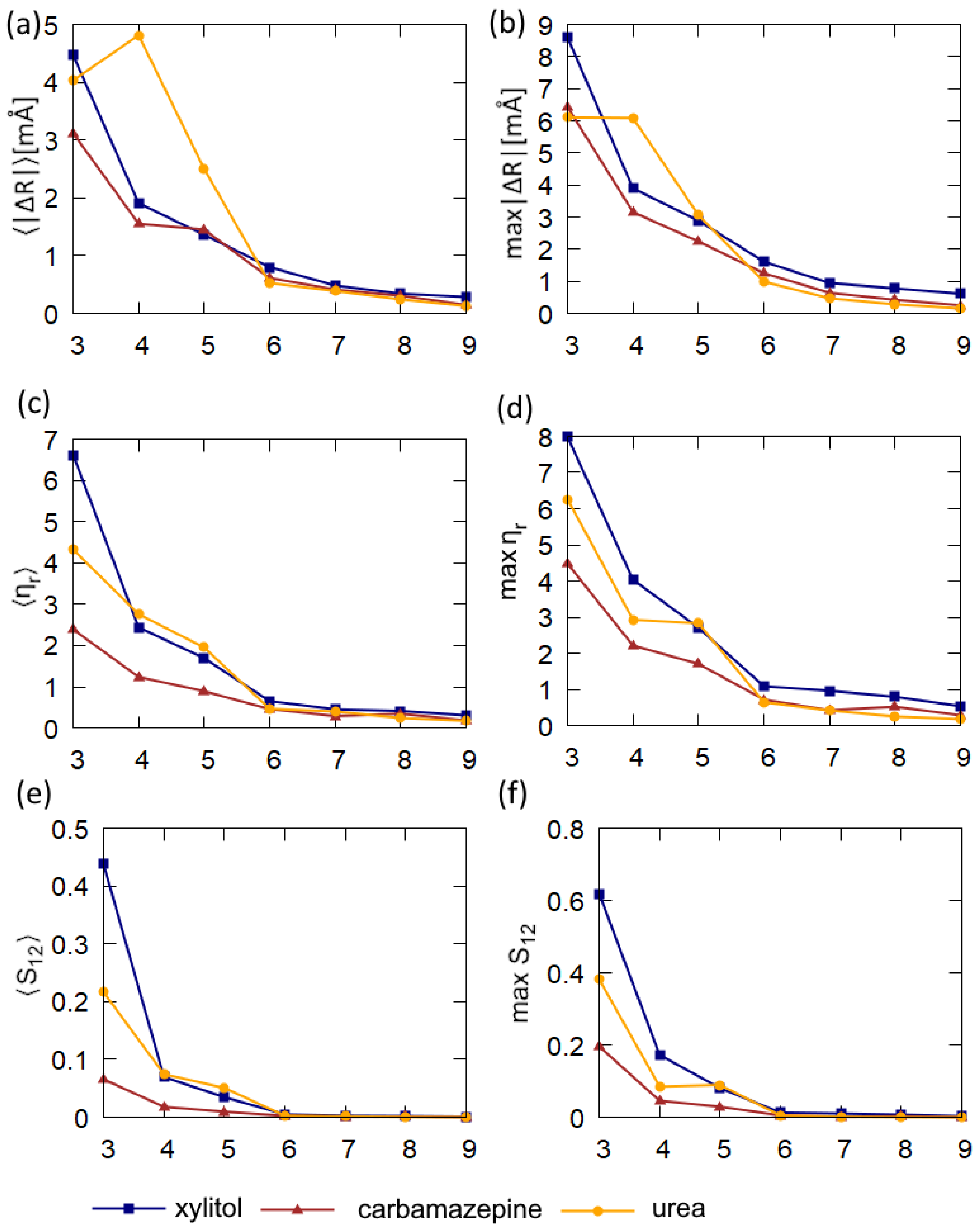

Figure S2 Convergence of structure obtained from HAR refinement with multipole expansion of the atomic electron densities (up to L_{max} order of spherical harmonics) to result from HAR without such an expansion in terms of (a) average and (b) maximum absolute difference in X-H bond lengths (c) average and (d) maximum values of rescaled overlapping index η_r for hydrogen atom ADPs and (e) average and (f) maximum S_{12} similarity index for hydrogen atom ADPs.

S4. Databank generation details

S4.1. Initial choice of structures from Cambridge Structural Database (CSD)

Initial set of structures is chosen using CSD search with the following criteria: structure (1) does not contain first or second group metal (2) heaviest permitted element – Ar (3) deposited after 2015 (4) R-factor<4.5% (5) exclude: disorder, errors, powder structures (6) C-C bond e.s.d. < 0.005Å (7) measurement temperature below 130K

S4.2. Final selection of molecules/ions

After each structure is divided into separate chemical units (molecules/ions) and atom types are assigned a **selection algorithm** is applied to select final set of chemical units for further use in the databank creation. This set is used as a source of input geometry for wave function calculations after an adjustment of X-H bond lengths to tabularized values from neutron measurements.

The chemical units **selections algorithm**:

A chemical unit **selection procedure** is applied repeatedly. It selects one chemical unit to the final set of chemical units. The procedure is repeated till all atom types are represented in at least **N** chemical units.

The chemical unit **selection procedure**:

1. Calculate **score function** for all chemical units not selected so far (the selected ones are not used in selection procedure).
2. Select the chemical unit with the highest score.

The **score function** calculation:

Score is calculated using the following expression:

$$s = \frac{1}{n\delta}\sum_{t} (N - n_t) * (0.1 - s_t)$$

where the summation runs over atom types, n is a number of atoms in the chemical unit, δ is 100 if there is a chemical unit with identical structural formula already chosen and 1 if it is not, t is an index of an atom type, N is a target number of chemical units containing atoms of a given type, n_t is a number of chemical units containing atom of a given type (t) selected so far, s_t is **similarity** to the other selected chemical units containing atom of type (t). Structural formulas comparison involved in evaluation of δ is performed with graph isomorphism algorithm, molecular graphs with chemical elements as ‘colours’ of graph nodes and chemical bonds as edges are used.

The **similarity** s_t is calculated using the following expression:

$$s_t = \frac{1}{n_t} \sum_k \frac{(f_k, f)}{\sqrt{(f_k, f_k)(f, f)}}$$

where the summations runs over chemical units containing atoms of type t selected so far. $s_t \in (0,1)$, (f_1, f_2) is a "scalar product" of chemical formulas:

$$(f_1, f_2) = \sum_e n(e, f_1) n(e, f_2)$$

where the summation runs over all chemical elements e occurring the two chemical formulas f_1 and f_2, $n(e, f_i)$ is number of atoms of the element e in the formula f_i.

S5. Comparison between neutron and X-ray refinement parameters related to hydrogen atoms

Table S3 Comparison of average difference in X-H bond lengths (in mÅ) between results from X-ray structure refinement and reference neutron study data ($R_{X-ray} - R_{neutron}$). HAR (±) stands for HAR with crystal environment represented via point multipoles, HAR (alone) for the version without such representation.

Model	TAAM	THAM	THAM	HAR	HAR (±)	THAM	HAR (±)
Method	B3LYP					Hartree-Fock	
Basis set	G-31G(d,p)		cc-pVTZ				
C-H							
Carbamazepine	-16.5	-9.2	-5.0	-4.2	-2.7	0.9	2.5
Gly ala	-2.9	1.8	3.6	5.0	5.6	10.5	1.3
NAC·H2O	-32.4	-21.2	-19.8	-18.2	-14.6	-11.8	-6.9
Xylitol	-2.8	-4.2	-3.5	-8.2	-3.7	3.8	3.8
O-H, N-H							
Carbamazepine	-44.5	-34.8	-30.0	-26.1	-18.6	-12.0	-2.9
Gly ala	-31.7	-17.0	-17.0	-16.9	-3.7	-2.3	11.1
NAC·H2O	-71.3	-51.2	-58.8	-52.1	-40.1	-31.0	-16.7
Urea	-18.5	-16.3	-9.9	-9.9	1.6	-5.1	9.6
Xylitol	-50.9	-33.0	-35.1	-29.6	-17.0	-7.0	9.0

Table S4 Comparison of hydrogen atom ADPs obtained with aspherical atom model X-ray refinements in terms of similarity index S_{12}, neutron diffraction experiments used as a reference. HAR (±) stands for HAR with crystal environment represented via point multipoles, HAR (alone) for the version without such representation.

Model	TAAM	THAM	THAM	HAR	HAR (±)	THAM	HAR(±)
Method	B3LYP					Hartree-Fock	
Basis set	G-31G(d,p)		cc-pVTZ				
H bonded to C							
Carbamazepine	2.78	1.3	1.12	0.99	0.99	1.68	1.63
Gly ala	2.08	1.76	1.57	1.93	1.6	2.28	2.47
NAC·H_2O	6.88	4.38	4.48	3.37	3.45	4.58	3.87
Xylitol	3.23	2.03	1.80	2.02	1.93	1.95	2.77
H bonded to O or N							
Carbamazepine	5.17	4.06	4.62	4.12	3.19	4.14	3.12
Gly ala	3.11	2.05	2.25	1.91	1.63	2.56	2.04
NAC·H_2O	14.10	9.33	10.68	8.83	6.26	10.0	8.18
Urea	1.24	1.08	1.32	1.79	0.75	2.61	1.03
Xylitol	6.54	3.84	5.36	5.44	3.00	4.67	2.81

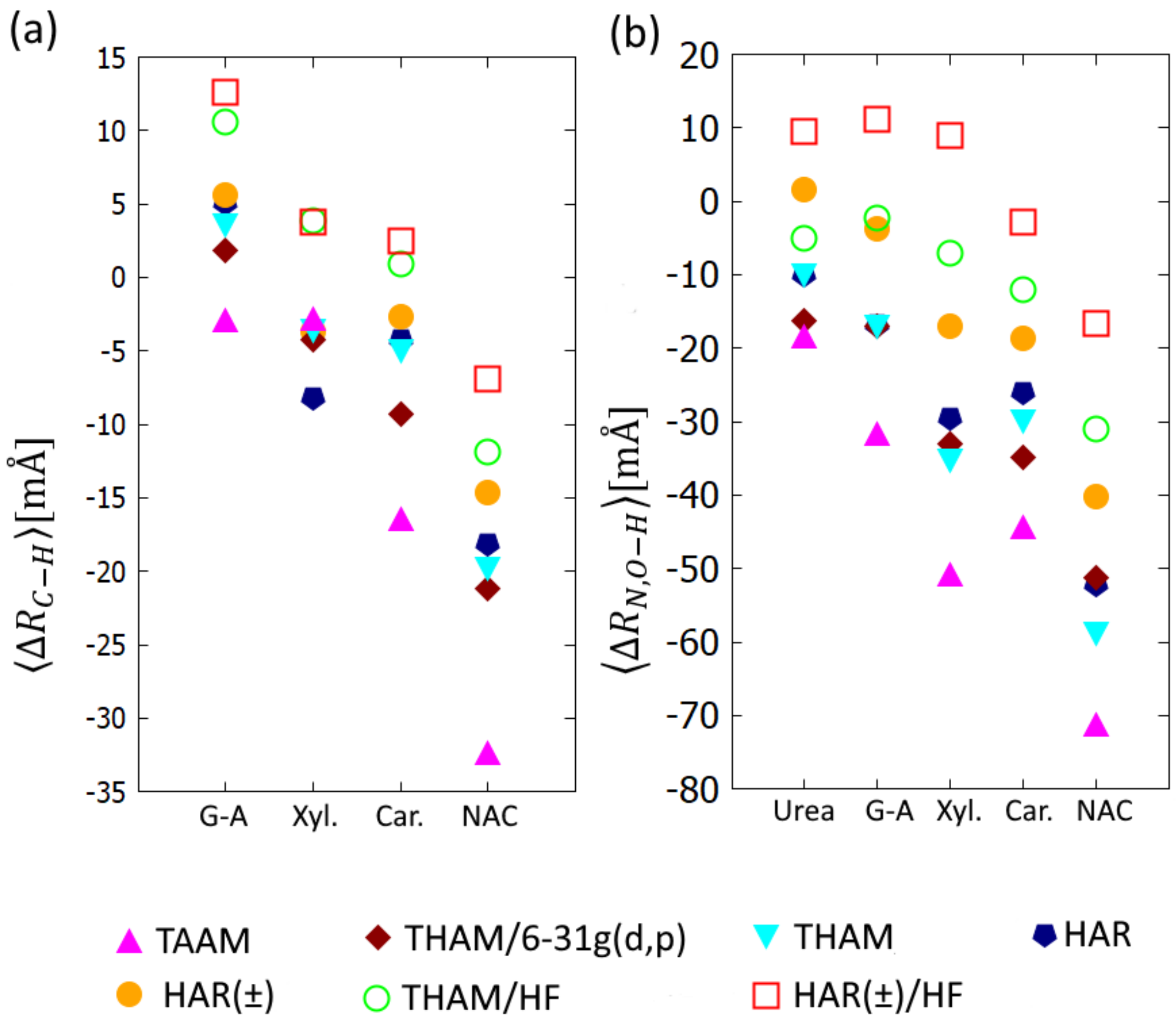

Figure S3 Comparison of: X-H bond lengths with reference neutron diffraction data in terms of average difference (in mÅ) for (a) non-polar (C-H) and (b) polar (N-H and O-H) bonds. THAM and HAR models are based on B3LYP/cc-pVTZ unless specified otherwise, (±) stands for HAR with crystal environment represented via point multipoles. Structure abbreviations: G-A - Gly–L-Ala, Xyl. – Xylitol, Car. – Carbamazepine, NAC - NAC·H2O.

Table S5 Comparison of R_1 and wR_2 agreement factors, aspherical atom models derived X-H bond lengths with reference neutron diffraction data in terms of absolute difference (in mÅ) hydrogen atom ADPs obtained with aspherical atom model X-ray refinements in terms of rescaled overlapping index η_r. All methods compared (TAAM, THAM and HAR with crystal environment represented via point multipoles) based on B3LYP/6-31g(d,p) level of theory.

Model	TAAM	THAM	HAR(±)	TAAM	THAM	HAR(±)
		R_1			wR_2	
Carbamazepine	2.62	2.64	2.63	6.61	6.65	6.33
Gly–L-Ala	1.56	1.52	1.51	3.16	3.04	2.97
NAC·H2O	2.46	2.42	2.39	4.91	4.80	4.74
Urea	1.56	1.50	1.50	4.06	3.90	3.97
Xylitol	1.71	1.67	1.64	3.18	3.07	3.00
$\langle\lvert\Delta R\rvert\rangle$		C-H			N,O-H	
Carbamazepine	17.5	11	8.9	44.5	34.8	25.8
Gly–L-Ala	8.1	9.1	5.0	31.7	17.0	6.4
NAC·H2O	32.4	21.2	16.8	71.3	51.2	31.1
Urea	-	-	-	18.5	16.2	7.3
Xylitol	9.4	11.8	8.3	50.9	33.0	14.6
$\langle\eta_r\rangle$		C-H			N,O-H	
Carbamazepine	15.5	10.2	10.1	21.9	19.4	17.2
Gly–L-Ala	12.4	12.3	12.4	16.1	13.1	12.5
NAC·H2O	24.0	19.3	17.4	36.1	29.0	22.8
Urea	-	-	-	11.0	10.0	10.1
Xylitol	16.6	12.7	12.8	23.8	19.0	13.6
